# Type 1 Diabetes in a Resource-Poor Setting: Malnutrition Related, Malnutrition Modified, or Just Diabetes?

**DOI:** 10.1007/s11892-018-1003-7

**Published:** 2018-06-14

**Authors:** Shitaye Alemu Balcha, David I.W. Phillips, Elisabeth R. Trimble

**Affiliations:** 1 Gondar College of Medicine and Health Sciences, Gondar, Ethiopia; 2 Medical Research Council’s Lifecourse Epidemiology Unit, Southampton General Hospital, Southampton, UK; 3 Centre for Public Health, Queen’s University Belfast, Belfast, UK

**Keywords:** Malnutrition-related diabetes, Type 1 DM, Resource-poor countries, Epidemiology, Undernutrition

## Abstract

**Purpose of Review:**

Very little is known about the occurrence of type 1 diabetes (T1DM) in resource-poor countries and particularly in their rural hinterlands.

**Recent Findings:**

Studies of the epidemiology of T1DM in Ethiopia and similar countries in sub-Saharan Africa show that the pattern of presenting disease differs substantially from that in the West. Typically, the peak age of onset of the disease is more than a decade later with a male excess and a low prevalence of indicators of islet-cell autoimmunity. It is also associated with markers of undernutrition.

**Summary:**

These findings raise the question as to whether the principal form of T1DM seen in these resource-poor communities has a different pathogenesis. Whether the disease is a direct result of malnutrition or whether malnutrition may modify the expression of islet-cell autoimmunity is unclear. However, the poor prognosis in these settings underlines the urgent need for detailed clinical and epidemiological studies.

## Introduction

There is a lack of good clinical and epidemiological studies of type 1 diabetes (T1DM) in resource-poor settings. The neglect is particularly evident in sub-Saharan Africa despite increasing recognition that T1DM is a significant and widespread health problem associated with unacceptably high rates of morbidity and mortality [1•]. Much of what we know in this part of the world is based on small-scale reports, often centred on urban areas. The data that do exist have suggested a disease pattern very unlike that found in the West, with an older age of clinical onset and the widespread occurrence of atypical forms [2••, 3•]. Furthermore, most of sub-Saharan Africa’s population still live in rural areas, and even less is known about the epidemiology of T1DM in these communities where traditional diets and lifestyle tend to be the norm together with food insecurity and under- rather than overnutrition. Studies of diabetes in these areas are particularly difficult to carry out because of the lack of medical staff, appropriate infrastructure and specialist laboratory analyses.

## Type 1 Diabetes in a Resource-Poor Setting

We have studied T1DM in Ethiopia, a country which has experienced many severe famines and in which more than 50% of children have evidence of nutritional stunting. Chronic undernutrition is common not only during intrauterine life and early childhood but also during the entire period of growth and adult life [4]. These studies together with recent similar studies in comparable populations suggest that there are major differences in the age and gender of presenting cases together with marked urban-rural variations in incidence. Importantly, they also suggest that there are differences in the role that autoimmunity plays in the pathogenesis of the disease, and highlight a possible contribution of undernutrition.

Studies on T1DM have been undertaken for more than a decade in two Ethiopian university centres, Gondar and Jimma, 750 km northwest and 330 km southwest of the capital, Addis Ababa, respectively. Each university hospital is linked to several clinics deep in the surrounding rural areas: these clinics consult with and are overseen by health care staff from the university centres. In this way, it has been possible to assess disease incidence, including T1DM, and evaluate differences between town and country. A study involving these two university centres in Ethiopia gave interesting results with respect to age of onset and differences between urban and rural areas.

**Fig. 1:**
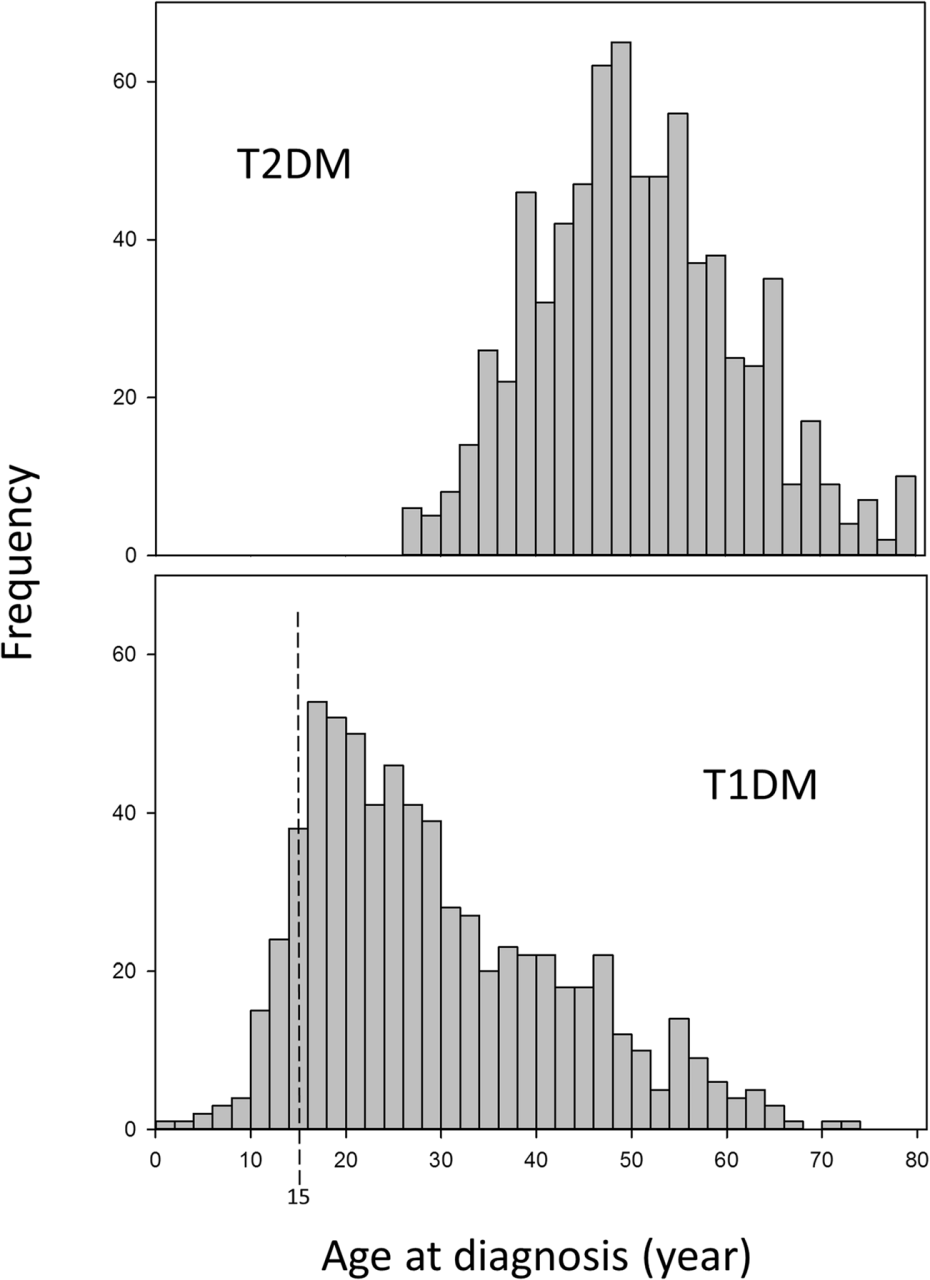
Age frequency of a series of 2280 patients with diabetes presenting at Gondar and Jimma hospitals, Ethiopia, 1995–2008. (Based on ref. 5). T1DM type 1 diabetes, T2DM type 2 diabetes

We found very low incidence of TIDM in childhood (3.3% diagnosed under 15 years) followed by a rapid increase in the mid-teens up to the age of 30 [5•]. This is illustrated in Fig. 1 which shows the age of presentation of a consecutive series of over 2280 diabetic patients over a 14-year period; nearly a half of the patients had T1DM. There were similar findings in a third culturally distinct area of Ethiopia, Mekelle [6], South Africa [7] and Cameroon [8]. By contrast, the pattern of disease reported from diabetes registries in the West show a much higher incidence rate in the under-15 age group (50–60%) with peak incidence at or before puberty [9]. As the rural areas of Ethiopia and other developing countries are exceptionally remote it is possible that some cases, especially in young children, may die undiagnosed. However, this is unlikely to be so in the urban areas where paediatric and diabetes services have been established for many years. For example, Kalk and colleagues in South Africa have reported a low incidence of childhood T1DM from a mainly urban population of Soweto with well-established paediatric medical services [7]. Another striking feature of the disease in Ethiopia was the male excess. Overall, the annual incidence in men was twice as high as that in women with the male excess more marked in the 15- to 40-year age group (sex ratio 2.4:1) than in those diagnosed at younger than 15 years (sex ratio 1.2:1). We have also found differences in incidence between urban and rural areas. In Ethiopia, urban centres had higher incidence rates than the surrounding rural areas [5•]; the annual urban rates were 11.9 and 6.1 per 100,000 for men and women, respectively, rates that did not differ much from Swedish figures for the 15–34 age group, the age group in which most of the Ethiopian cases occur [9]. By contrast, the corresponding rates for the rural communities were 2.2 and 1.0 per 100,000 for men and women, respectively. More than 90% of the population live in rural areas so, despite the lower incidence rate, the total number of T1DM cases in the rural areas was more than twice the number in the urban areas—an important fact for infrastructure planning for rural communities.

Of particular interest is the effect of transition to a more western lifestyle which has been studied in Jews who have emigrated to Israel from Ethiopia; a large number originated from the rural areas around Gondar, an area included in our survey of T1DM [10•]. Not only was there an increase in the incidence of T1DM to one of the highest in Israel but also, in those carrying two high-risk susceptibility genes for T1DM, there was a reduction in the age of onset which correlated with the time the family had lived in Israel before the birth of the diabetic subject [11]. This suggests that lifestyle has an effect on the epidemiology of T1DM within Ethiopia and exposure to environmental factors (nutritional and other) causes a disease transition to a ‘Western pattern’ of diabetes with an earlier onset. This is supported by the finding that migrants to Sweden whose parents came from east Africa showed an increased incidence of T1DM [12].

## A Role for Undernutrition?

In a case-control study in Ethiopia, we showed that T1DM was strongly associated with poverty and markers of undernutrition, most marked in the rural cases. Affected patients reported a history of childhood malnutrition, and the loss of the patient’s mother during early childhood; this was coupled with evidence of disproportionate skeletal growth, especially in men, and other evidences of poverty and overcrowding [10•]. However, the clearest evidence for undernutrition as an aetiological factor in diabetes comes from animal studies. Reductions in total food intake during pregnancy or early postnatal life lead to decreased glucose tolerance and diabetes in the offspring. The rodent maternal protein-restricted model is one of the most extensively studied. Low-protein-fed (5–8%) dams give birth to growth-restricted offspring and when suckled by the same low-protein-fed dams during lactation, they remain permanently growth-restricted, despite being weaned on to a control (20% protein) diet. This dietary manipulation approximates to the levels of protein nutrition observed in developing countries [13]. Undernutrition at particular times in pregnancy affects the methylation and alters the activity of many genes that control hepatic and pancreatic function. These epigenetic changes alter genes that control growth and transcription factors important for pancreatic development and β-cell differentiation such as IGF2 [14], PDX1 [15] and HNF4-α [16], resulting in a permanently reduced β-cell mass and reduced insulin secretion. Altered HNF4-α function (gene mutations or deletions) in humans has also been associated with maturity onset diabetes of the young, a non-obese form of diabetes that can be mistaken for T1DM [17]. Deficiencies in the postnatal diet can also affect IGF2 imprinting in mice and lead to reduced IGF2 levels [14]. Changes in micronutrient levels alone in the peri-conceptual period can alter the methylation of many genes, even when the caloric intake is maintained. In sheep, altered levels of micronutrients that contribute to the methylation process such as vitamin B_12_, folic acid and methionine have been shown to change the methylation status of many genes in the liver and pancreas; it is of interest that more than 50% of the affected loci are specific to males [18]. In humans, epidemiological evidence for an effect of early undernutrition on the development of diabetes has come from a study of men and women born during the Dutch Winter Famine of 1944–45 showing that gross total undernutrition during gestation resulted in poorer glucose tolerance and impaired insulin secretion when followed up 60 years later [19, 20].

Most studies have linked early undernutrition with insulin resistance and type 2 diabetes (T2DM), for example in South India where low BMI and low vitamin B_12_ status during pregnancy is associated with intrauterine growth retardation [21] and insulin-resistant diabetes in the offspring [22]. However, it is clear that T1DM can also occur and that the effects of early undernutrition are manifest as T1DM or T2DM according to the degree, timing and macro- or micronutrient specificity of the nutritional deficit. In the case of people living in urban areas of developing countries, a change to a western-type diet and the development of obesity would increase the risk of developing insulin-resistant T2DM.

## Islet-cell Autoimmunity in Sub-Saharan Africa

Autoimmunity is believed to be a very important factor in the aetiology of T1DM, especially that arising in childhood. Recent studies have reported rates of islet autoantibody positivity ranging from 30% in Tanzania [23], 29–35% in Ethiopia [24•, 25] to 43% in Cameroon [8] in T1DM. These studies have been carried out for the most part in adult patients and some in mainly urban areas. It is known that in Caucasian patients, the rates of autoantibody positivity in T1DM decrease with age of onset in newly diagnosed cases; typical values for autoantibody positivity are 85–98% positive with onset at the age of 15 years and under [26], 76% positive with onset at 15–34 years [27] and 67% with onset at 34 years and above [28]. These results suggest that, even when using age-appropriate comparisons, the proportion of diabetes resulting from autoimmunity in sub-Saharan Africa may be lower than in the European population. Recent reports from Cameroon [8], Tunisia [29] and Egypt [30] have drawn attention to the influence of HLA status on autoantibody production in T1DM in these countries. In regions where there are lower frequencies of diabetes-associated HLA haplotypes, there may be fewer cases of diabetes with an autoimmune basis, although this is unlikely to be the only factor. This is an area that requires further study in order to define more clearly the role of autoimmunity in the aetiology of T1DM in other areas of Africa.

The low rates of autoimmunity in sub-Saharan Africa may be due to a higher proportion of the patients having non-immunogenic diabetes as a result of impaired pancreatic β-cell development as described above. However, it is also possible that the reduced propensity to islet-cell autoimmunity reflects the effects of chronic undernutrition on the immune system. In the Gambia, severe malnutrition in subsistence farmers occurs for several months each year (the ‘hungry’ season) before the next harvest. Children born in the ‘hungry’ season show evidence of intrauterine growth retardation and have a smaller thymus than those born after harvest; they also have a 10-fold increased likelihood of death from infectious causes in early adult life, suggesting a permanent impairment of the immune system [31–33]. In Ethiopia, many dietary constituents are involved in chronic undernutrition and it is likely that more than one could be involved in modulating the immune system. For example, almost half the stunted children had zinc deficiency [34]. In one study, more than 30% of the mothers were protein-calorie deficient, 20% were vitamin A deficient, and many had low levels of zinc in their breast milk [35]; zinc [36] and vitamin A [37] deficiencies are known to alter the immune response. In the Gambia, mothers who give birth in the ‘hungry’ season have less IL-7 in their breast milk than those who give birth when food is more plentiful [38]; IL-7 is essential for the proliferation and survival of precursor T cells [39]. Since the fetal thymus releases T cells as early as 14 weeks gestation, any nutritional insult acting before or at that time could have lasting effects on the immune responses. Clearly, the specifics of maternal and in utero malnutrition vary geographically in the developing world; however, in Ethiopia, there are nutritional deficiencies that, theoretically, could give rise to immune deficiencies and altered autoimmune responses in later life, and may contribute to the low incidence of childhood T1DM.

## Conclusions

Recent epidemiological studies of T1DM in sub-Saharan Africa suggest that the common disease pattern differs from that in the West with a later age of onset, a male excess at presentation and a lower prevalence of indicators of islet-cell autoimmunity together with markers of undernutrition (Table 1). These features have a striking similarity to previous clinical descriptions of malnutrition-related diabetes [40, 41], which at one time was recognised as a separate entity by the World Health Organization. They suggest a need to clarify the role of nutrition or related aetiological factors affecting the presentation of T1DM in resource-poor settings. A key question is whether the disease seen in these communities is a direct result of malnutrition and the consequent reduction of pancreatic β-cell number and function, a result of malnutrition influencing the expression of islet-cell autoimmunity or, more likely, a combination of both processes. These studies are needed to help address the very great morbidity and mortality of patients with T1DM in poor communities and improve their diagnosis and treatment.

**Table 1:** Features of T1DM in resource-poor compared with western settings

Resource poor	Western
Uncommon in children, peak incidence 25–29 years	Incidence rises to a peak at age 10–14 years
Male excess	Genders equally affected in under 15 age group
Associations with markers of undernutrition	Associated with increased childhood growth
Prevalence of islet-cell antibodies low	High prevalence of islet-cell antibodies
Major urban-rural differences in incidence	No consistent urban-rural pattern
